# Temperature Variations on the External Root Surface During Warm Injectable Gutta-Percha Obturation at Different Thermo-Plasticization Settings: An In Vitro Study

**DOI:** 10.3390/medicina62061159

**Published:** 2026-06-15

**Authors:** Mihai Paven, Adrian-George Marinescu, Osama Abuabboud, Laura-Elena Cîrligeriu, Luminița-Maria Nica, Bianca Georgiana Cărăbiș, Izabella Maria Kovacs, Oana-Alexandra Velea-Barta, Roxana Oancea

**Affiliations:** 1 Department of Restorative Dentistry and Endodontics, Research Center TADERP, Faculty of Dentistry, “Victor Babes” University of Medicine and Pharmacy Timisoara, 300041 Timisoara, Romania; mihai.paven@umft.ro (M.P.); marinescu.adrian@umft.ro (A.-G.M.); osama.abuabboud@umft.ro (O.A.); cirligeriu.laura@umft.ro (L.-E.C.); 2 Faculty of Dentistry, “Victor Babes” University of Medicine and Pharmacy Timisoara, 300041 Timisoara, Romania; dr.carabisbianca@gmail.com; 3 Department of Restorative Dentistry and Endodontics, Faculty of Dentistry, “Victor Babes” University of Medicine and Pharmacy Timisoara, 300041 Timisoara, Romania; izabella.kovacs@umft.ro (I.M.K.); velea.oana@umft.ro (O.-A.V.-B.); 4 Translational and Experimental Clinical Research Centre in Oral Health, Department of Preventive, Community Dentistry and Oral Health, Faculty of Dentistry, “Victor Babes” University of Medicine and Pharmacy Timisoara, 300041 Timisoara, Romania; roancea@umft.ro

**Keywords:** gutta-percha, root canal obturation, thermography, temperature, endodontics

## Abstract

*Background and Objectives*: Warm injectable gutta-percha techniques may improve three-dimensional filling of complex canal anatomy, but heat transfer to the external root surface may threaten periodontal tissues when the 47 °C threshold is exceeded. This in vitro study quantified external root-surface temperature changes during obturation with the Woodpecker FI-G/FI-P system and compared manufacturer preset temperatures with actual device output. *Materials and Methods*: Twenty extracted single-rooted human teeth standardized to 18 mm were prepared and assigned to obturation at 180 °C (Group A, *n* = 10) or 230 °C (Group B, *n* = 10). Infrared thermography recorded coronal, middle, and apical root-surface temperatures. A second device-based experiment measured FI-G and FI-P output at preset temperatures of 150 °C, 180 °C, 200 °C, and 230 °C. *Results*: The 230 °C setting produced significantly higher middle-third temperatures than the 180 °C setting (41.84 ± 5.52 °C vs. 36.99 ± 3.21 °C; *p* = 0.027; Cohen’s d = 1.07), whereas coronal and apical differences were not significant. The highest external root-surface value was 49.6 °C, and 3/10 specimens obturated at 230 °C exceeded 47 °C in the middle third. A significant coronal-to-middle gradient reversal was observed at 230 °C (*p* = 0.045). Device measurements showed strong attenuation between preset and output temperatures: at 230 °C, the FI-G tip base reached 150.0 °C but the tip apex reached 51.3 °C, while FI-P plugger tips reached 120.0 °C. *Conclusions*: The 180 °C setting produced a more predictable thermal profile, whereas 230 °C increased localized middle-third overheating risk. These in vitro findings support cautious temperature selection, especially in teeth with reduced dentin thickness or compromised root anatomy.

## 1. Introduction

It is well established in the endodontic literature that numerous dental procedures generate heat that may exert detrimental effects on the dental pulp and surrounding periodontal structures [1]. Various in vitro investigations have quantified temperature elevations at the pulp and root surface level during different stages of endodontic treatment, including canal preparation, irrigation, and obturation [1,2]. The thermal behavior of these procedures has become an important focus of research, as exceeding critical temperature thresholds can lead to irreversible tissue damage, including bone necrosis and periodontal ligament destruction [3].

Endodontic treatment success is fundamentally dependent on achieving a complete, three-dimensional, and hermetic seal of the root canal system following thorough chemomechanical debridement [4,5]. Among the various obturation techniques available, warm gutta-percha injection methods have gained popularity due to their ability to adapt to complex root canal anatomies, improving the quality of canal filling and reducing voids [6]. These techniques involve heating gutta-percha to temperatures ranging from 160 °C to 200 °C or higher, which allows the material to flow into lateral canals, isthmuses, and irregularities that cold lateral condensation techniques may not adequately fill [7].

From a clinical perspective, external root-surface heating is relevant because periodontal ligament cells, cementum, and adjacent alveolar bone may be exposed to heat transmitted through dentin during obturation. The risk is not determined by device preset alone, but by the interaction between temperature, exposure duration, residual dentin thickness, root morphology, and the presence of anatomical defects or resorptive areas [3,8,9].

Several factors influence the magnitude of heat transfer to the root surface during warm obturation procedures, including the thickness of residual dentin, the cross-sectional configuration of the root canal, the volume and temperature of injected gutta-percha, and the duration of heat application [1,2]. Lipski reported that root surface temperature elevations in mandibular incisors, where residual dentin is thin, reached up to 22.1 °C when using gutta-percha heated to 160 °C with the Obtura II system [2]. In contrast, maxillary central incisors demonstrated temperature increases of approximately 8.5 °C under identical conditions, highlighting the critical role of dentin thickness as a thermal insulator [10,11].

Accordingly, the scientific gap addressed by the present study concerns the lack of quantitative data on whether different thermoplasticization presets of the Woodpecker FI-G/FI-P system produce clinically relevant differences in external root-surface temperature, and whether nominal device presets correspond to the temperatures actually delivered at the active instrument endpoints [12,13,14,15]. This distinction is important because device presets may overestimate or underestimate the heat transmitted to dentin and the external root surface.

The null hypothesis was that the 180 °C and 230 °C thermoplasticization settings would not produce significant differences in external root-surface temperatures at the coronal, middle, or apical thirds, and that the measured device output would not meaningfully differ from the manufacturer-indicated preset temperatures.

Despite the extensive literature on temperature variations during warm obturation, limited data exist regarding the thermal performance of newer injection systems at different preset temperature levels, and few studies have systematically compared manufacturer-specified temperatures with actual recorded values or characterized the directionality and magnitude of intra-root thermal gradients. The Woodpecker FI-G/FI-P system represents a contemporary obturation platform composed of an injectable gutta-percha gun and heated pluggers, both of which may contribute to thermal transfer during clinical use [8,10]. Therefore, the present in vitro investigation was intentionally designed as two complementary studies under the same manuscript title: a tooth-based study evaluating external root surface temperature variations during obturation at 180 °C and 230 °C, and a device-based study assessing the actual temperatures generated by the FI-G gun with the E23G injection cannula and by the FI-P heated pluggers (5506 and 5508) across the available presets. A secondary aim was to interpret both datasets together in order to determine whether the thermal output recorded directly on the devices could explain the regional heating patterns observed on the external root surface.

## 2. Materials and Methods

### 2.1. Study Design and Sample Selection

This in vitro experimental investigation comprised two complementary study arms performed under the same thermal imaging protocol. The initial experiment (Study 1) used 20 extracted single-rooted human permanent teeth and a comparative design with two experimental groups defined by the FI-G injection temperature setting: Group A (180 °C, *n* = 10) and Group B (230 °C, *n* = 10). Study 2 evaluated the real thermal output of the obturation devices themselves, namely the Woodpecker FI-G gun fitted with the E23G injection cannula and the Woodpecker FI-P heated electronic pluggers (types 5506 and 5508), across all four manufacturer-defined temperature presets (150 °C, 180 °C, 200 °C, and 230 °C). The purpose of structuring the work in this manner was to correlate the clinically relevant external root-surface findings with the actual temperatures recorded directly on the obturation instruments.

The study was approved by the Scientific Research Ethics Committee of the Victor Babeș University of Medicine and Pharmacy Timișoara (Approval number 19 from 22 March 2024) and was conducted in accordance with the principles of the Declaration of Helsinki. Teeth were obtained from routine dental extractions performed for clinical reasons unrelated to the study. Written informed consent for research use of the extracted teeth was obtained before collection, and all specimens were anonymized immediately after extraction.

Because this was an exploratory in vitro thermal study and preliminary data for the specific FI-G/FI-P protocol were unavailable, a formal a priori power calculation was not feasible. The sample size of 20 teeth (10 per group) was selected based on specimen availability, ethical constraints related to the use of extracted human teeth, and comparability with previous laboratory studies evaluating thermal changes during obturation. The sample size should therefore be interpreted as adequate for detecting large thermal differences, while smaller differences may have been missed.

Strict inclusion criteria were applied to ensure sample homogeneity. Teeth were required to be permanent, single-rooted, with fully formed apices, straight roots without pronounced curvature, and similar dimensions. Exclusion criteria comprised multi-rooted teeth, teeth with open apices, calcified canals, primary teeth, teeth with pronounced root curvature, and teeth with atypical anatomy or structural defects such as root fractures or previous endodontic treatment. All selected teeth were sectioned to a standardized length of 18 mm to eliminate variability related to root length.

Before allocation, each specimen was inspected visually and under magnification to exclude cracks, external resorption, root caries, previous endodontic treatment, and structural defects. Buccolingual and mesiodistal root dimensions were checked with a digital caliper at the coronal, middle, and apical thirds to avoid extreme anatomical variability. The 18 mm standardized length allowed the external root surface to be divided into equal 6 mm thirds; thermographic regions of interest were placed at the center of each third to ensure reproducible coronal, middle, and apical measurements. Residual dentin thickness was not quantified by CBCT or micro-CT, and this was considered in the interpretation of inter-specimen variability.

### 2.2. Sample Preparation Protocol

Following extraction, all teeth underwent thorough cleaning to remove calculus, soft tissue remnants, and debris from the root surfaces. Each tooth was sectioned to 18 mm in length using a diamond disc under water cooling. The prepared specimens were stored in physiological saline solution throughout the study to maintain hydration and simulate clinical conditions as closely as possible within the constraints of an in vitro model.

All 20 teeth were mechanically prepared using the ProTaper Next nickel-titanium rotary system (Dentsply Sirona, Ballaigues, Switzerland) following the manufacturer’s recommended sequence. During preparation, sodium hypochlorite (NaOCl) was used as the irrigant. After completion of chemomechanical preparation, the teeth were placed in numerically labeled sponge-type supports for systematic identification and arranged into two groups of 10 specimens each.

The working length was established 1 mm short of the apical foramen, and all canals were prepared using the same final instrumentation protocol. Irrigation was standardized for all specimens using 2.5% sodium hypochlorite between instruments, followed by a final rinse with 17% EDTA and physiological saline. Canals were dried with sterile paper points before obturation. The same irrigation sequence, solution volume, and drying procedure were used in both experimental groups to reduce procedural variability.

### 2.3. Obturation Procedure and Temperature Measurement in the Two Complementary Studies

In Study 1, root canal obturation was performed using the Woodpecker FI-G injectable gutta-percha system (Guilin Woodpecker Medical Instrument Co., Ltd., Guilin, China). For Group A, the injection system was set to a thermoplasticization temperature of 180 °C, while Group B was obturated at the 230 °C setting. Both settings represent two of the four available temperature presets on the device. The model E23G injection cannula (0.6 mm diameter and 28 mm length), which is the dedicated delivery tip for the backfilling phase of the Woodpecker FI-G obturation system, was used for all obturation procedures. The injectable filling material consisted of Meta Biomed EQ-V gutta-percha bars (Meta Biomed Co., Ltd., Cheongju, Republic of Korea), selected for compatibility with the induction-heating mechanism of the system. Each tooth was positioned in a metallic vise-type holder during obturation to ensure stable positioning and a constant distance from the thermal imaging device.

For thermal standardization, no endodontic sealer was used; this allowed the experiment to isolate the temperature effect of the thermoplasticized gutta-percha and device setting without introducing variability related to sealer type, quantity, or thermal conductivity. The E23G cannula was inserted to approximately 3 mm short of the working length and withdrawn coronally at a constant rate while gutta-percha was injected. Each specimen received a single injection cycle, and the activation/injection time was standardized to 10 s. The device was allowed to return to the selected preset temperature before each specimen was obturated.

All obturation procedures and thermographic recordings were performed by one calibrated operator with endodontic training. Prior to data acquisition, the operator completed pilot procedures on non-study teeth to standardize hand position, cannula depth, withdrawal speed, and camera alignment. This single-operator design minimized interoperator variability, although it also limited the ability to evaluate interoperator reliability.

Temperature measurements were obtained in real time using the built-in thermovision camera of a Blackview BV8900 smartphone (Blackview, Shenzhen, China), equipped with a FLIR Lepton 3.5 thermal imaging sensor. For Study 1, temperature was recorded at the coronal, middle, and apical thirds of the external root surface, together with the ambient temperature. In Study 2, direct thermal measurements were performed on the FI-G gun at three standardized locations (main body, base of the heating tip, and tip apex) for all four presets (150 °C, 180 °C, 200 °C, and 230 °C). In parallel, the FI-P heated electronic pluggers type 5506 and type 5508, each with a 0.55 mm tip diameter and 6% and 8% taper, respectively, were assessed at the tip, middle third, and base under the same preset conditions. The plugger measurements were included because these components are used clinically for searing off excess gutta-percha and softening the material during vertical compaction, and their actual output needed to be interpreted in relation to the temperatures measured on teeth.

**Figure 1:**
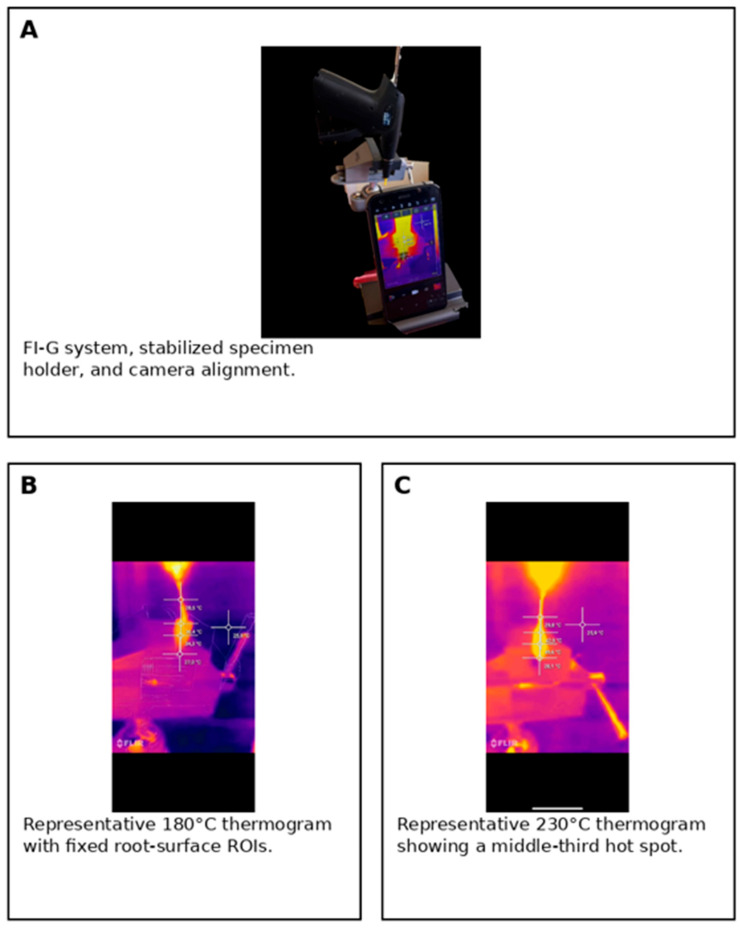
Thermovision-based experimental setup and measurement landmarks used for Study 1. (**A**) Woodpecker FI-G injection system, stabilized specimen holder, and thermal camera alignment. (**B**,**C**) Representative thermograms illustrating fixed coronal, middle, and apical regions of interest used to extract external root-surface temperatures during obturation. ROls = regions of interest at coronal, middle, and apical thirds.

The thermal camera was positioned at a fixed distance from the external root surface, and the tooth, holder, and camera were not moved during each recording. The coronal, middle, and apical regions of interest were defined according to the three equal 6 mm root segments after standardization to 18 mm. The highest temperature visible within each predefined region during the injection cycle was recorded. Ambient temperature was recorded for each specimen to confirm environmental stability across groups (Figure 1).

### 2.4. Statistical Analysis

For Study 1, descriptive statistics including minimum, maximum, mean, and standard deviation were calculated for all root-surface and ambient measurements. One-way analysis of variance (ANOVA) was used for between-group comparisons at each root third, with significance set at α = 0.05. The Mann–Whitney U test was employed as a non-parametric alternative for thermal gradient comparisons given the non-normal distribution of gradient data. For Study 2, the FI-G and FI-P recordings were summarized descriptively at each preset and measurement location, and the resulting output profile was interpreted alongside the root-surface dataset in order to identify whether attenuation at device level paralleled the thermal behavior observed on teeth.

Pearson correlation coefficients were computed to evaluate the strength and significance of linear relationships between temperatures at different root thirds within each group. Cohen’s d effect sizes with 95% confidence intervals were calculated for all between-group comparisons to quantify the magnitude of temperature differences independent of sample size. Effect sizes were classified as small (|d| < 0.5), medium (0.5 ≤ |d| < 0.8), or large (|d| ≥ 0.8). The coefficient of variation (CV%) was computed to assess measurement reproducibility and inter-specimen variability. The CV ratio (Group B/Group A) was used to evaluate whether higher injection temperatures amplify specimen-to-specimen temperature variability.

Normality assumptions were evaluated descriptively because of the small sample size. The Mann–Whitney U test was retained for gradient comparisons because these values were not normally distributed. Formal interoperator reliability testing was not applicable because acquisition and region-of-interest extraction were performed by a single calibrated operator; therefore, measurement reproducibility and variability were reported through the coefficient of variation and CV ratio. This limitation is explicitly acknowledged in the Discussion.

## 3. Results

The raw temperature data from all 20 specimens are presented in Table 1. In Group A (180 °C preset), root surface temperatures at the coronal third ranged from 31.5 °C (specimens A2 and A5) to 42.4 °C (specimen A4). The middle third ranged from 32.1 °C to 40.8 °C, while the apical third showed consistently lower values between 24.4 °C and 31.1 °C. In Group B (230 °C preset), the middle third exhibited the highest individual temperature of any measurement at 49.6 °C (specimen B7), the only value exceeding the critical 47 °C threshold. Three specimens in the middle third of Group B (B4 at 47.3 °C, B7 at 49.6 °C, and B9 at 47.2 °C) exceeded this threshold, representing 30% of the group. Ambient temperatures were stable across both groups, ranging from 22.5 °C to 27.1 °C, confirming consistent environmental conditions.

The descriptive analysis (Table 2) reveals important thermal patterns. In Group A, the coronal and middle third means were nearly identical (36.79 ± 4.25 °C vs. 36.99 ± 3.21 °C), suggesting uniform heat distribution. The apical third showed a substantial drop to 27.49 ± 2.25 °C. In Group B, the middle third displayed the highest mean (41.84 ± 5.52 °C), surpassing the coronal third (36.56 ± 4.45 °C) by 5.28 °C—a gradient reversal phenomenon not observed in Group A. The SD in the middle third of Group B was the highest (5.52 °C), indicating greater variability likely attributable to differences in residual dentin thickness and canal morphology among specimens.

The ANOVA results (Table 3) demonstrate region-specific statistical differences. The coronal third comparison yielded F = 0.014 (*p* = 0.907), indicating no significant difference. In the middle third, F = 5.770 exceeded F-critical = 4.41, yielding a significant *p*-value of 0.027, confirming the 230 °C setting generates significantly higher middle-third temperatures. The apical third showed a borderline result (F = 3.269, *p* = 0.087). The region-specific nature of these findings underscores that global comparisons would mask the localized thermal risk concentrated in the middle third.

The Study 2 characterization of the FI-G gun (Table 4) revealed substantial discrepancies between preset and actual temperatures. At the 150 °C preset, the tip base reached only 70.4 °C and the tip apex 35.4 °C, whereas at the 230 °C preset the tip base reached 150.0 °C and the tip apex 51.3 °C. Thus, even at the highest nominal setting, the active terminal segment of the device recorded far lower values than the programmed temperature, while the main body remained comparatively stable (61.6–79.1 °C), supporting effective insulation. When these data are interpreted together with Study 1, they help explain why root-surface temperatures stayed well below the nominal device presets, yet clinically relevant hot spots could still emerge in the middle third when the 230 °C mode was used.

Both FI-P models generated substantial tip temperatures that increased proportionally with the preset (Table 5), confirming that the plugger component should also be regarded as part of the thermal environment of warm vertical compaction. Maximum tip values of 120.0 °C were reached at the 230 °C preset for both models, whereas the middle third and base remained within an approximate 30–40 °C interval, demonstrating a steep and localized thermal gradient concentrated at the working end. At 180 °C, the P5508 reached 102.1 °C compared with 95.2 °C for the P5506, while at 230 °C both converged at the same maximum tip temperature. These findings complement Study 1 by showing that the system delivers high focal temperatures at the instrument tip, but that this heat is rapidly attenuated away from the active end, which is consistent with the lower temperatures recorded on the external root surface.

The clinical safety analysis (Table 6) shows that in Group A (180 °C), no specimens exceeded 47 °C in any root third. The maximum was 42.4 °C at the coronal third, 4.6 °C below the critical threshold. In Group B (230 °C), three specimens (B4, B7, B9) in the middle third exceeded 47 °C, representing 30%. The mean temperature rise in the middle third of Group B was +4.84 °C above body temperature; below the critical 10 °C mean increase, the maximum individual temperature of 49.6 °C (specimen B7) represents a 12.6 °C rise, exceeding the critical threshold. These data suggest that the 230 °C setting carries meaningful risk in the middle third, particularly with thinner dentin walls.

The intra-root thermal gradient analysis (Table 7) provides a novel perspective by quantifying temperature differentials between adjacent root regions. The coronal-to-middle gradient revealed a striking between-group difference: in Group A, this gradient was negligible (−0.20 ± 2.16 °C), indicating nearly uniform heat distribution. In Group B, a substantial negative gradient (−5.28 ± 5.67 °C) indicates the middle third was on average 5.28 °C hotter than the coronal third—a thermal gradient reversal phenomenon. This difference was statistically significant by Mann–Whitney U test (U = 77.0, *p* = 0.045), suggesting the 230 °C setting causes gutta-percha to retain sufficient thermal energy to create a paradoxical temperature peak deeper in the canal. The middle-to-apical gradient was larger in Group B (12.90 ± 6.16 °C) compared to Group A (9.50 ± 2.73 °C), though not significant (*p* = 0.212). The total coronal-to-apical gradient was surprisingly smaller in Group B (7.62 °C) than Group A (9.30 °C), reflecting the mid-third thermal peak.

The Pearson correlation analysis (Table 8) reveals fundamentally different thermal coupling patterns between groups. In Group A, a strong and highly significant positive correlation was observed between the coronal and middle thirds (r = 0.869, *p* = 0.001), indicating specimens with higher coronal temperatures also showed proportionally higher middle-third temperatures—consistent with a predictable, monotonic thermal decay model. The coronal-to-apical correlation was moderate but non-significant (r = 0.568, *p* = 0.086). In Group B, all correlations were substantially weaker and non-significant: coronal-middle (r = 0.370, *p* = 0.293), coronal-apical (r = −0.150, *p* = 0.680), and middle-apical (r = −0.472, *p* = 0.168). The loss of inter-regional thermal coupling in Group B, particularly the negative correlations, suggests that at 230 °C, thermal behavior becomes non-linear and unpredictable, governed more by local anatomical factors than by systematic proximal-to-distal heat decay.

The effect size analysis (Table 9) provides a sample-size-independent assessment. The middle third exhibited a large effect size (d = 1.074, 95% CI [0.137, 2.012]), with the CI excluding zero, confirming a robust difference. The apical third also showed a large effect (d = 0.809) but its CI crossed zero. The coronal-to-middle gradient comparison yielded the largest effect (d = −1.185, 95% CI [−2.135, −0.235]), CI excluding zero, confirming the gradient reversal as the most robust finding. The CV analysis revealed Group B showed substantially higher middle-third variability (13.20%) compared to Group A (8.67%), a CV ratio of 1.52, indicating the 230 °C setting amplifies inter-specimen variability by 52%. In contrast, apical CV was lower in Group B (4.02% vs. 8.20%), CV ratio 0.49, suggesting more uniform apical temperatures at higher settings—possibly due to thermal equilibration at the apex regardless of injection temperature.

**Figure 2:**
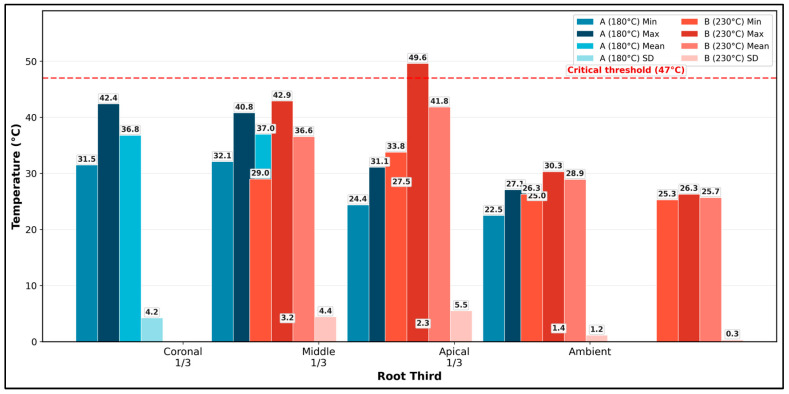
Temperature distribution by root third and experimental group: minimum, maximum, mean, and standard deviation with data labels. The dashed red line indicates the critical 47 °C threshold.

Figure 2 presents the comparative temperature distributions for all measurement locations. In Group A (blue bars), mean values were 36.8 °C and 37.0 °C for coronal and middle thirds, respectively, with maxima of 42.4 °C and 40.8 °C. The apical third showed substantially lower temperatures (mean 27.5 °C, max 31.1 °C), well below the 47 °C threshold. In Group B (red bars), the middle third maximum of 49.6 °C was the only value exceeding the threshold, with a mean of 41.8 °C. The SD bars confirm that Group B’s middle third (5.5 °C) displays 72% greater variability than Group A’s middle third (3.2 °C). Ambient temperatures remained stable and nearly identical between groups (A: 25.0 ± 1.4 °C; B: 25.7 ± 0.3 °C).

**Figure 3:**
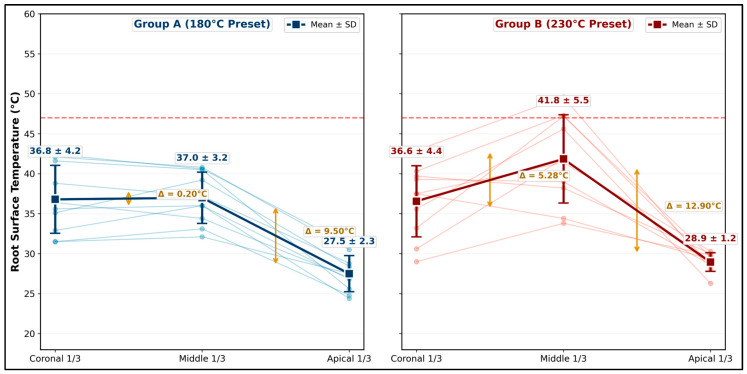
Intra-root thermal gradient profiles showing per-specimen decay trajectories (thin lines) and mean ± SD (bold line with error bars). Gold arrows indicate mean inter-third temperature differentials (Δ).

**Figure 4:**
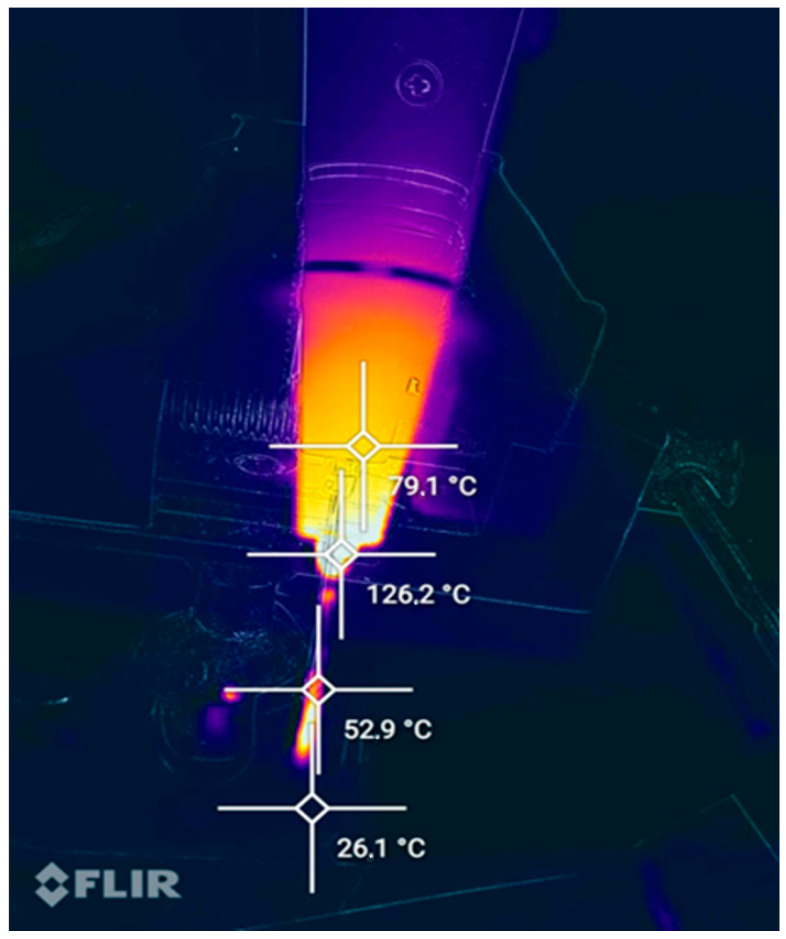
Representative FI-G injection system thermogram at the 200 °C preset.

**Figure 5:**
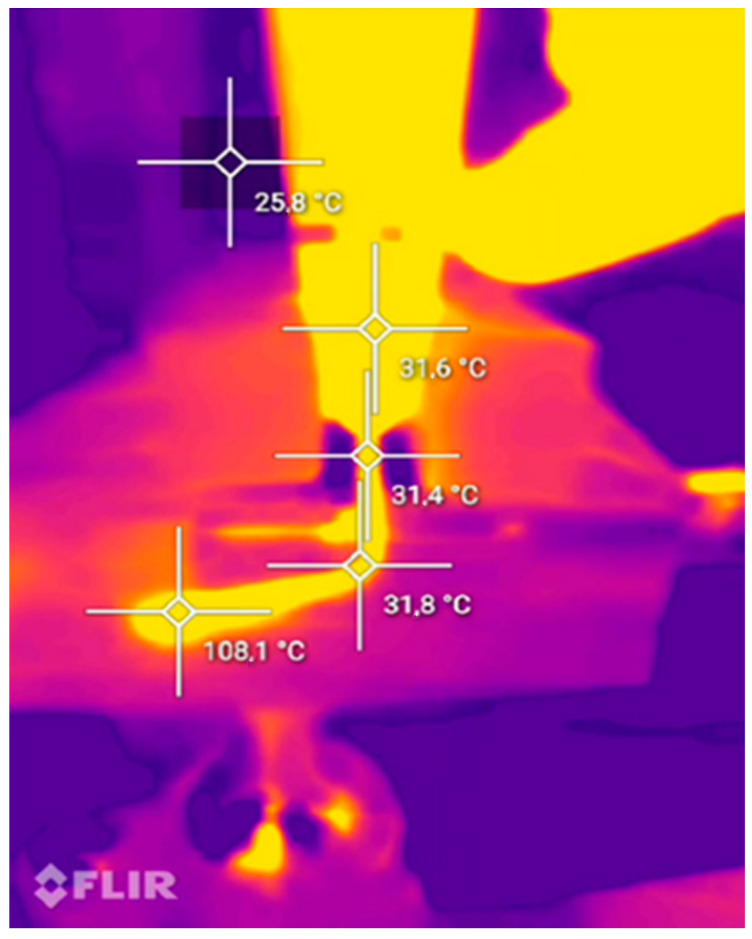
Representative FI-P plugger 5508 thermogram at the 200 °C preset.

Figure 3 illustrates the per-specimen thermal decay trajectories from coronal to apical thirds. In Group A (left panel), specimens show a monotonic decay—temperature remains stable between the coronal (36.8 ± 4.2 °C) and middle thirds (37.0 ± 3.2 °C), with a minimal gradient of 0.20 °C, followed by a steep 9.50 °C drop to the apical third (27.5 ± 2.3 °C). Trajectories are parallel and clustered, reflecting the strong r = 0.869 correlation. In Group B (right panel), trajectories exhibit a non-monotonic inverted-V shape, with individual lines diverging in the middle third where several specimens exceed 47 °C. The mean coronal-to-middle gradient was −5.28 °C, indicating paradoxical temperature increase, followed by a 12.90 °C descent to the apical third (28.9 ± 1.2 °C), as seen in Figure 4 and Figure 5. The wider spread in Group B’s middle third corresponds to the CV of 13.20%.

**Figure 6:**
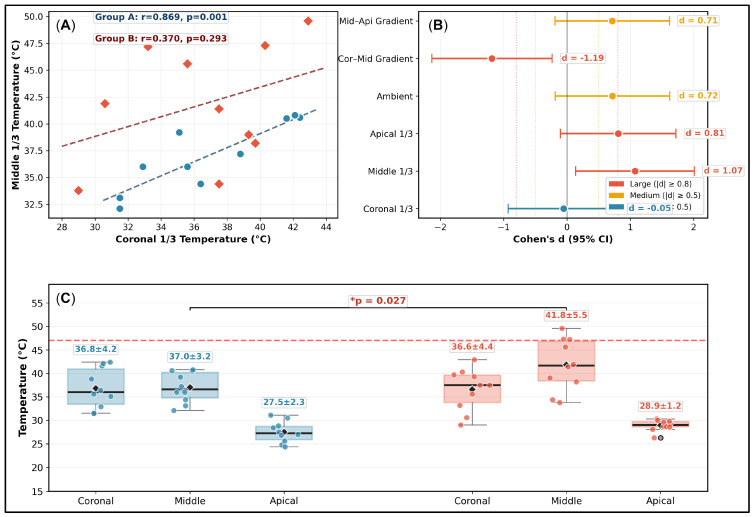
Multi-panel statistical analysis. (**A**) Scatter plot with Pearson correlation and regression lines. (**B**) Forest plot of Cohen’s d effect sizes with 95% CI. (**C**) Box-and-jitter plot with significance bracket; * = statistically significant.

**Figure 7:**
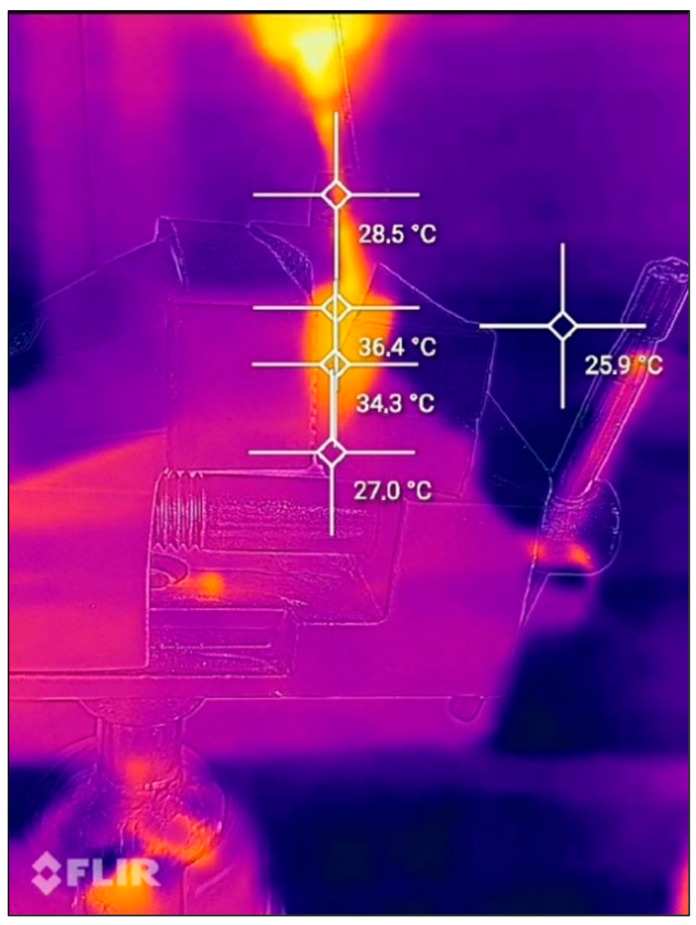
Representative infrared thermography image of specimen A1 during obturation at 180 °C.

**Figure 8:**
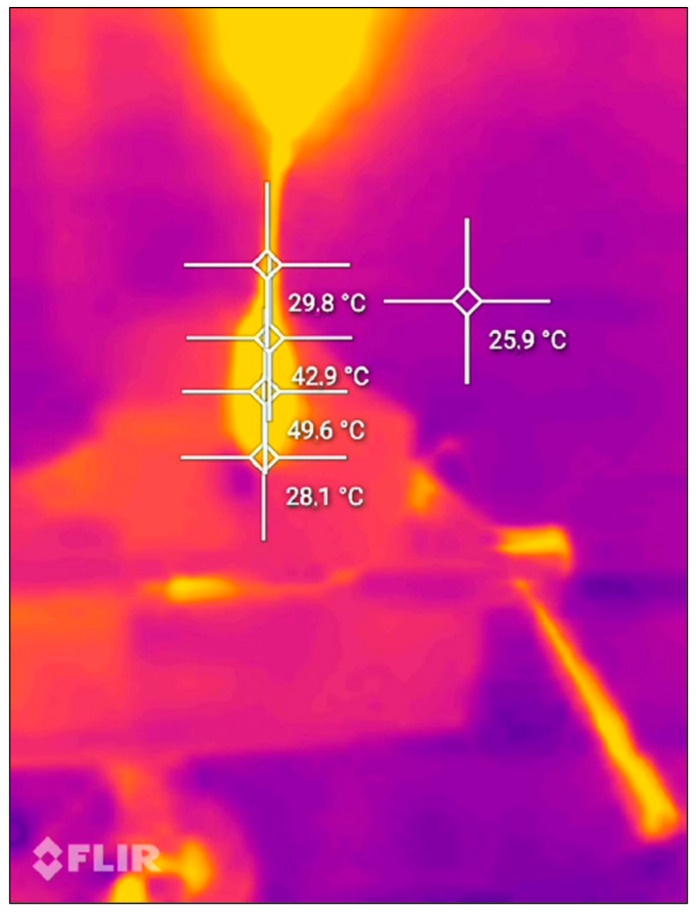
Representative infrared thermography of specimen B7 during obturation at 230 °C, showing the highest recorded temperature (49.6 °C).

Figure 6 integrates correlation, effect size, and distributional analyses. Panel A demonstrates Group A’s tight positive coronal-middle relationship (r = 0.869, *p* = 0.001) versus Group B’s dispersed, non-significant pattern (r = 0.370, *p* = 0.293), indicating thermal uncoupling at 230 °C. Panel B identifies the coronal-to-middle gradient reversal as the largest effect (d = −1.19), followed by the middle third (d = 1.07), both with CIs excluding zero. The apical third (d = 0.81) and mid-apical gradient (d = 0.71) have CIs crossing zero. Panel C shows box plots with individual data points, the significance bracket over the middle third (*p* = 0.027), and the 47 °C threshold line illustrating three Group B specimens penetrating the danger zone (Figure 7 and Figure 8).

## 4. Discussion

### 4.1. Analysis of Findings

The principal contribution of this manuscript is that it integrates two complementary thermal studies performed on the same obturation platform. Study 1 demonstrated that the 230 °C injection setting produced significantly higher temperatures in the middle third (*p* = 0.027, Cohen’s d = 1.07), while no significant differences were observed in the coronal or apical thirds. Beyond absolute comparisons, the thermal gradient analysis revealed a gradient reversal phenomenon in Group B, where the middle third was paradoxically hotter than the coronal third (Δ = −5.28 °C vs. −0.20 °C; *p* = 0.045), constituting the largest effect size in the study (d = −1.19). Study 2 strengthens the interpretation of these findings by showing that the FI-G and FI-P devices do not deliver their nominal preset temperature uniformly to the clinically relevant endpoints, but rather display marked attenuation and localized concentration of heat. Taken together, the two study arms suggest that the external root-surface response reflects both preset selection and the non-linear manner in which thermal energy is transferred from the obturation instruments to dentin and gutta-percha.

The clinical interpretation of these findings requires caution. The 47 °C threshold is commonly used as a reference for potential periodontal and osseous injury, but tissue response depends on both temperature and exposure time. A short transient peak may not have the same biological effect as sustained heating, and an in vitro thermogram cannot directly quantify cellular injury. Therefore, the present data should be interpreted as evidence of relative thermal risk between settings rather than proof of clinical harm or complete clinical safety.

An important finding from the complementary device analysis was the substantial discrepancy between manufacturer-preset and actual recorded temperatures. At the 230 °C setting, the FI-G tip base reached 150.0 °C whereas the tip apex recorded 51.3 °C, and the FI-P pluggers reached 120.0 °C at the tip while remaining much cooler at the middle and base. These observations indicate that preset values should not be interpreted as synonymous with the temperature experienced throughout the working parts of the obturation system. Chang et al. [14] reported significant time-dependent plugger temperature variations, and Dimopoulos et al. [16] found notable differences between commercial heating devices. Fioretti et al. [15] likewise demonstrated progressive temperature increases with repeated activations. In the present study, the joint interpretation of Studies 1 and 2 suggests that although thermal attenuation contributes to overall safety at the root surface, it does not eliminate the possibility of localized overheating when higher presets are selected.

The observed middle-third hot spot at 230 °C is biologically plausible but should not be overinterpreted. Heat transfer during warm obturation is influenced by residual dentin thickness, canal diameter, the proximity of the gutta-percha mass to the external root surface, and the duration of device activation. Thin dentin may reduce thermal insulation, whereas thicker root walls and surrounding tissues may dissipate heat more effectively. Because dentin thickness was not measured three-dimensionally in this study, the gradient reversal should be regarded as an experimental observation that requires confirmation in larger samples and in different tooth types [2,7,11,12,13,17,18,19,20].

Compared with previous reports on warm vertical compaction, injectable gutta-percha, and thermoplasticized obturation systems, the present results support the concept that most heat is attenuated before reaching the external root surface, but that localized elevations can still occur under higher settings [2,3,7,11,12,13,17,18,19,20]. Differences among studies may reflect variations in tooth type, residual dentin thickness, thermocouple versus infrared measurement techniques, device design, activation duration, ambient conditions, and whether simulated periodontal tissues or surrounding media were used. These methodological differences explain why some studies report modest thermal changes while others identify potentially critical root-surface elevations.

The findings are most relevant to higher-risk clinical situations, including teeth with thin root walls, immature apices, extensive canal enlargement, internal or external resorption, root concavities, previous iatrogenic damage, or compromised periodontal support. In such cases, even transient middle-third heating may be more clinically relevant because reduced dentin thickness decreases the insulating barrier between the warmed filling material and periodontal tissues. Clinicians should therefore consider using lower temperature settings, shorter activation times, intermittent heating, and careful control of the depth and duration of the delivery tip in anatomically compromised teeth. Nevertheless, these findings should be interpreted in light of potential residual confounding from unmeasured or incompletely controlled factors, including underlying comorbidities and other patient- and treatment-related characteristics [21,22,23,24,25,26,27,28,29].

### 4.2. Limitations and Future Research

Several limitations should be explicitly recognized. First, the in vitro design did not reproduce the periodontal ligament, alveolar bone, blood flow, or adjacent soft tissues. These structures may dissipate heat in vivo, whereas the isolated extracted-tooth model may either overestimate or alter the spatial distribution of external root-surface temperatures. Conversely, the absence of biological tissues means that the study cannot determine whether a given thermal peak would actually cause periodontal or osseous injury.

Second, the sample size was limited to 20 single-rooted teeth. Although the middle-third difference was statistically significant and associated with a large effect size, the study was not powered to detect small or moderate differences at every root level. The borderline apical comparison and the wide variability observed at the 230 °C setting should therefore be interpreted cautiously.

Third, anatomical variability could not be eliminated completely. Dentin thickness, canal cross-section, root concavities, and microscopic structural differences were not quantified using CBCT or micro-CT. These factors probably contributed to the higher middle-third variability in Group B and may explain why only some specimens exceeded the 47 °C threshold.

Fourth, all procedures were performed by a single calibrated operator. This reduced interoperator variability but prevented formal interoperator reliability analysis. Future studies should include multiple calibrated operators, duplicate thermogram readings, and formal intra- and interobserver reliability testing.

Fifth, only single-rooted teeth and two injection settings were evaluated in the tooth-based experiment. Future projects should include multi-rooted teeth, roots with reduced dentin thickness, simulated periodontal ligament and bone, different obturation systems, repeated activation cycles, and direct comparison between thermography and thermocouple measurements. These additions would strengthen clinical extrapolation and help define safer operating parameters for compromised anatomies.

## 5. Conclusions

Within the limitations of this in vitro investigation, the 180 °C setting produced a lower and more predictable external root-surface thermal profile than the 230 °C setting. The 230 °C setting generated significantly higher middle-third temperatures and was associated with a localized gradient reversal, with 3/10 specimens exceeding the 47 °C reference threshold. Direct device measurements confirmed that manufacturer presets did not correspond to uniform output temperatures at the active endpoints, as heat was concentrated near the working tips and attenuated rapidly away from them. These findings support cautious use of higher thermoplasticization settings and suggest that lower settings, shorter activation times, and careful control of delivery-tip depth may be preferable in teeth with thin dentin, resorptive defects, or compromised root anatomy. Because the study was performed in vitro, the results should not be interpreted as definitive evidence of clinical safety or injury, and further studies using simulated periodontal tissues, larger samples, multi-rooted teeth, and formal reliability testing are warranted.

## Figures and Tables

**Table 1:** Temperature recordings (°C) at the three root thirds and ambient for all 20 specimens.

Group	ID	Coronal (°C)	Middle (°C)	Apical (°C)	Ambient (°C)
A	A1	36.4	34.4	27.0	25.9
A	A2	31.5	33.1	24.8	22.5
A	A3	41.6	40.5	25.6	23.6
A	A4	42.4	40.6	31.1	27.1
A	A5	31.5	32.1	27.5	25.5
A	A6	35.6	36.0	24.4	23.3
A	A7	35.1	39.2	28.4	25.5
A	A8	42.1	40.8	30.5	25.7
A	A9	38.8	37.2	28.8	25.5
A	A10	32.9	36.0	26.8	25.1
B	B1	30.6	41.9	28.7	25.6
B	B2	37.5	41.4	30.3	25.8
B	B3	35.6	45.6	26.3	26.3
B	B4	40.3	47.3	28.7	25.7
B	B5	37.5	34.4	29.3	25.4
B	B6	29.0	33.8	29.8	25.4
B	B7	42.9	49.6	28.1	25.9
B	B8	39.7	38.2	30.0	26.2
B	B9	33.2	47.2	29.6	25.4
B	B10	39.3	39.0	28.6	25.3

**Table 2:** Descriptive statistics of temperature recordings (°C) for Groups A and B.

Group	Location	Min	Max	Mean	SD
A (180 °C)	Coronal	31.5	42.4	36.79	4.25
	Middle	32.1	40.8	36.99	3.21
	Apical	24.4	31.1	27.49	2.25
	Ambient	22.5	27.1	24.97	1.40
B (230 °C)	Coronal	29.0	42.9	36.56	4.45
	Middle	33.8	49.6	41.84	5.52
	Apical	26.3	30.3	28.94	1.16
	Ambient	25.3	26.3	25.70	0.35

**Table 3:** One-way ANOVA comparison of root surface temperatures between Group A and Group B.

Root Third	Mean A	Mean B	F	*p*-Value	F-crit	Significance
Coronal 1/3	36.79	36.56	0.014	0.907	4.41	Not Significant
Middle 1/3	36.99	41.84	5.770	0.027	4.41	Significant
Apical 1/3	27.49	28.94	3.269	0.087	4.41	Not Significant

**Table 4:** Study 2: temperature measurements (°C) on the FI-G injection device at different manufacturer presets.

Preset (°C)	Main Body	Tip Base	Tip Apex	Mean
150	61.6	70.4	35.4	55.80
180	62.3	79.0	41.4	60.90
200	79.1	126.2	52.9	86.07
230	70.8	150.0	51.3	90.70

**Table 5:** Study 2: temperature measurements (°C) on FI-P plugger tips (models 5508 and 5506).

Plugger	Preset	Tip	Mid 1/3	Base	Ambient	Mean
P5508	150	82.6	36.3	31.2	27.1	50.03
	180	102.1	32.4	31.3	27.0	55.27
	200	108.1	31.8	31.6	25.8	57.17
	230	120.0	34.4	33.3	26.0	62.57
P5506	150	79.5	32.9	33.9	25.3	48.77
	180	95.2	30.2	35.9	25.9	53.77
	200	104.0	35.6	31.3	25.6	56.97
	230	120.0	39.7	32.5	25.4	64.07

**Table 6:** Analysis of specimens exceeding the critical 47 °C threshold and temperature rise above body temperature (37 °C).

Group	Root Third	n > 47 °C	% >47 °C	ΔT vs. 37 °C	Max (°C)	>10 °C Rise
A (180 °C)	Coronal	0/10	0%	−0.21	42.4	No
	Middle	0/10	0%	−0.01	40.8	No
	Apical	0/10	0%	−9.51	31.1	No
B (230 °C)	Coronal	0/10	0%	−0.44	42.9	No
	Middle	3/10	30%	+4.84	49.6	No
	Apical	0/10	0%	−8.06	30.3	No

**Table 7:** Intra-root thermal gradient analysis: temperature differentials (°C) between root thirds with Mann–Whitney U test.

Gradient Segment	A MeanΔ	A SD	B MeanΔ	B SD	U-Stat	*p*-Value (MWU)
Coronal → Middle	−0.20	2.16	−5.28	5.67	77.0	0.045
Middle → Apical	9.50	2.73	12.90	6.16	33.0	0.212
Coronal → Apical	9.30	3.50	7.62	4.76	60.0	0.473

**Table 8:** Pearson correlation matrix of temperatures between root thirds within each group.

Group	Pair 1	r	*p*-Value	Pair 2	r	*p*-Value
A (180 °C)	Cor-Mid	0.869	0.001	Cor-Api	0.568	0.086
	Mid-Api	0.547	0.102			
B (230 °C)	Cor-Mid	0.370	0.293	Cor-Api	−0.150	0.680
	Mid-Api	−0.472	0.168			

**Table 9:** Effect size analysis (Cohen’s d with 95% CI) and coefficient of variation (CV%) for between-group comparisons.

Comparison (B vs. A)	Cohen’s d	95% CI	Effect	CV% A	CV% B	CV Ratio
Coronal 1/3	−0.053	[−0.930, 0.824]	Negligible	11.55	12.17	1.00
Middle 1/3	1.074	[0.137, 2.012]	Large	8.67	13.20	1.52
Apical 1/3	0.809	[−0.103, 1.720]	Large	8.20	4.02	0.49
Ambient	0.717	[−0.187, 1.621]	Medium	5.61	1.36	0.24
Cor–Mid Gradient	−1.185	[−2.135, −0.235]	Large			
Mid–Api Gradient	0.714	[−0.190, 1.618]	Medium			

## Data Availability

The data presented in this study are available on request from the corresponding author.
